# Extracorporeal Membrane Oxygenation Support in a Young Patient With COVID-19 Infection

**DOI:** 10.7759/cureus.8694

**Published:** 2020-06-19

**Authors:** Gian Lima, Eduardo Cardoso, Maria Camila Paredes

**Affiliations:** 1 Internal Medicine, University of Connecticut Health Center, Farmington, USA

**Keywords:** ecmo, vv ecmo, covid, covid-19

## Abstract

Critically ill patients with coronavirus disease 2019 (COVID-19) infection often require mechanical ventilation, and still many of them will progress to worsening hypoxia and death. Veno-venous extracorporeal membrane oxygenation (VV-ECMO) has been used in some centers, but its role in the setting of COVID-19 infection is still unclear to date. We describe a case of a young female patient with obesity but otherwise no other underlying medical conditions who was admitted with respiratory failure secondary to COVID-19. Given her severe acute respiratory distress syndrome (ARDS) with refractory hypoxemia, she was treated with VV-ECMO. After a prolonged hospital course, she improved clinically and was able to have VV-ECMO explanted, after 18 days of extracorporeal therapy.

The complexity of ECMO therapy requires a well-trained multidisciplinary team present only at expert centers. The high resource cost is a challenge to the health care system in times of a global pandemic. Considering the limitations of this resource-intensive therapy, clinical judgment is crucial to decide whether ECMO is an appropriate option for the patient. However, for young patients with no underlying conditions who are critically ill despite optimized mechanical ventilation, we believe that extracorporeal therapy represents a reasonable option when available

## Introduction

The coronavirus disease 2019 (COVID-19) pandemic has overwhelmed health care systems around the world. Critically ill patients often require mechanical ventilation, and still many of them will progress to worsening hypoxia and death. For selected cases, veno-venous extracorporeal membrane oxygenation (VV-ECMO) has been used in some centers, but the unclear role of this therapy in the setting of COVID-19 infection, its high cost, and complexity represent a challenge for resource allocation in times of global pandemic. We describe a case of a young female patient with obesity but otherwise no other underlying medical conditions who was admitted with respiratory failure secondary to COVID-19. Given her severe acute respiratory distress syndrome (ARDS) with refractory hypoxemia, she was treated with VV-ECMO successfully.

## Case presentation

A 32-year-old female with a past medical history of obesity (body mass index of 42) and gastroesophageal reflux disease, not on home medications, presented to the emergency department complaining of worsening shortness of breath for two days. The patient was in her usual state of health until seven days before presentation, when she developed a dry cough, fever, chills, and malaise. She worked as a certified nursing assistant at a nursing home facility where a few cases of COVID-19 had been detected recently.

In the emergency department, her initial vital signs were as follows: temperature 99°F, pulse 106 beats per minute, blood pressure 107/70 mmHg, respiratory rate 34 breaths per minute, and oxygen saturation 70% on room air. Physical examination revealed an alert and oriented patient (Glasgow Coma Scale 15), with bilateral wheezing but otherwise unremarkable physical findings. She was promptly placed on nasal cannula and subsequently on nonrebreather 100% FiO_2_, with an improvement of her oxygen saturation to 95%.

Her initial laboratory results showed a white blood cell count of 10.9 K/µL (normal range [NR] 3.8-10.8 K/µL), absolute lymphocyte count of 0.64 K/µL (NR 0.85-3.9 K/µL), hemoglobin 14.1 g/dL (NR 13.2-17.1 g/dL), platelets 214 K/µL (NR 140-400 K/µL), C-reactive protein 30.94 mg/dL (NR < 0.80 mg/dL), ferritin 998 µg/L, lactic acid 3.3 mmol/L (NR 0.5-2.2 mmol/L), lactate dehydrogenase 556 U/L (NR 125-220 U/L), troponin was negative (NR < 0.30 ng/mL), creatinine 1.0 mg/dL (NR 0.7-1.3 mg/dL), Na 138 mmol/L (NR 135-145 mmol/L), K 3.4 mmol/L (NR 3.5-5.1 mmol/L), and high-sensitivity D-dimer 806 ng/mL (NR < 231 ng/mL).

**Figure 1:**
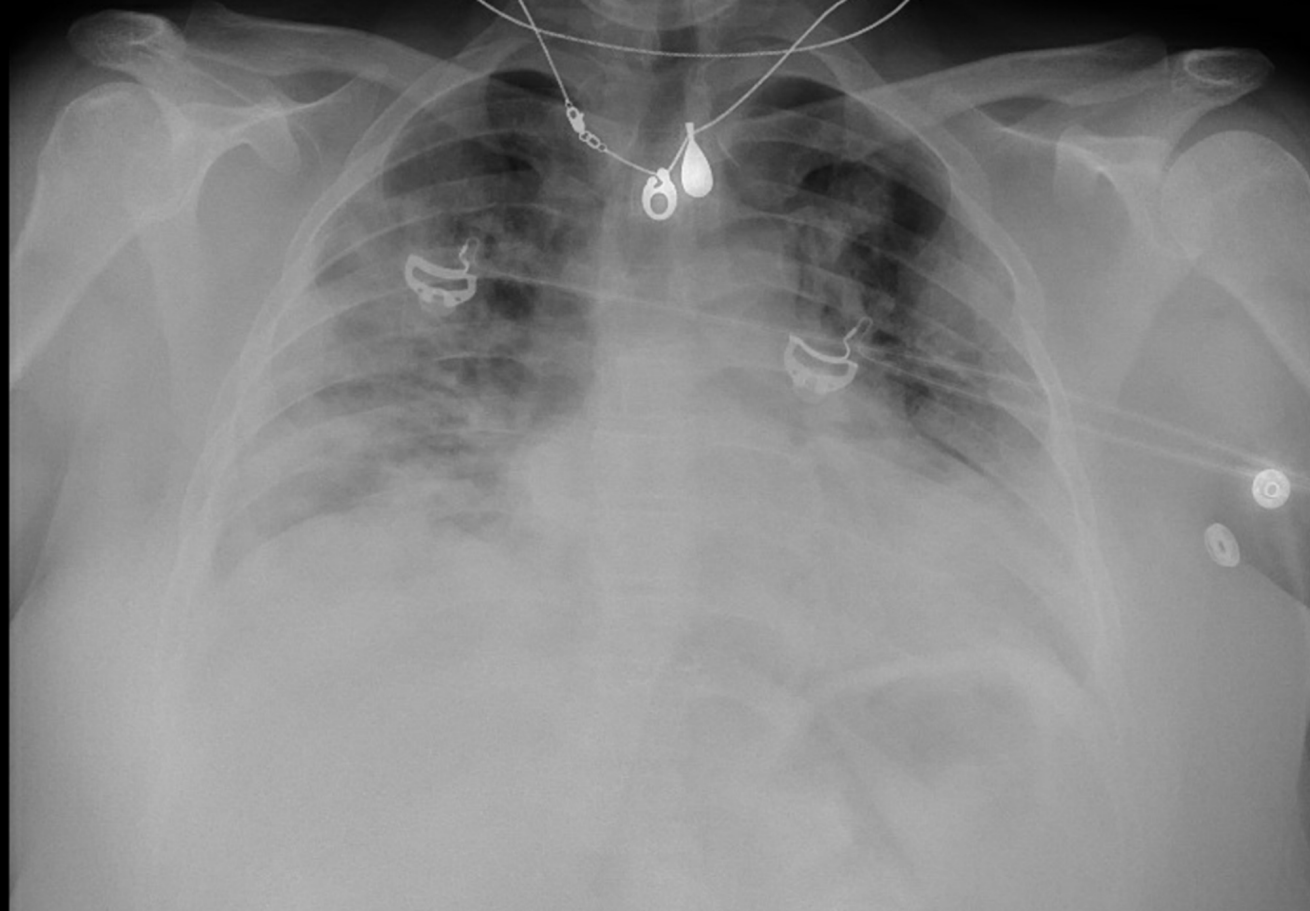
Chest radiograph Chest radiograph from admission was consistent with COVID-19 pneumonia, with multiple rounded and peripheral ground-glass opacities throughout the lung fields.

The chest radiograph obtained in the emergency department demonstrated multiple rounded and peripheral ground-glass opacities throughout the lung fields (Figure 1).

A nasopharyngeal swab was performed and tested positive for severe acute respiratory syndrome coronavirus 2 (SARS-CoV-2) RNA. Due to severe hypoxia, the patient was admitted to the intensive care unit (ICU). On hospital day 2, she underwent intubation and mechanical ventilation for worsening tachypnea and respiratory distress. The patient was started on intravenous steroids (methylprednisolone 40 mg every eight hours), received a dose of tocilizumab 400 mg, and completed a five-day course of hydroxychloroquine (800 mg/day for one day, then 400 mg/day for four days) and azithromycin (500 mg/day for one day, then 250 mg/day for four days).

**Figure 2:**
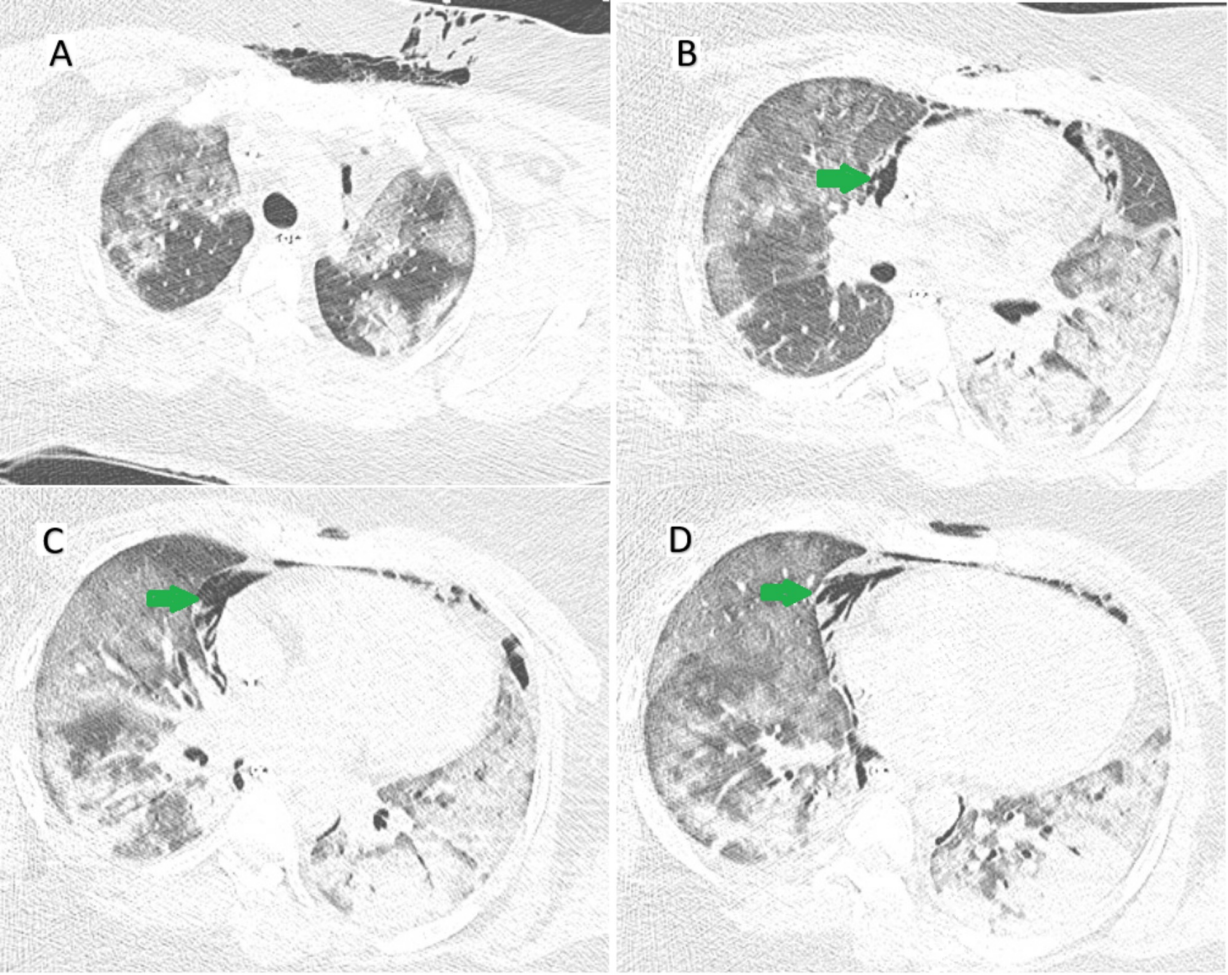
CT of the chest (A) CT of the chest demonstrates diffuse ground-glass airspace opacities throughout both lungs. This finding is commonly found in COVID-19 pneumonia. (B-D) The presence of extensive pneumomediastinum (green arrows), secondary to barotrauma from the mechanical ventilation.

On hospital day 3, her renal function worsened, and her creatinine peaked at 2.4 mg/dL, likely secondary to acute tubular necrosis. However, she remained with good urine output and no significant metabolic acidosis. Her creatinine gradually improved on the subsequent days, returning to the baseline renal function. The patient remained stable hemodynamically and sedated with hydromorphone and ketamine, but despite the optimized medical management she continued to spike fevers at 102°F, and repeated chest imaging with CT on day 5 showed significant worsening bilateral ground-glass airspace opacities (Figure 2).

On hospital day 7, she received convalescent plasma transfusion and started on vancomycin after respiratory cultures demonstrated the presence of Staphylococcus aureus infection. On hospital day 8, the patient had an episode of oxygen desaturation to 75% with a PaO_2_/FiO_2_ ratio of 79, despite being on mechanical ventilation with FiO_2_ 100% and positive end-expiratory pressure (PEEP) 12 cm H_2_O. She was bagged by the respiratory therapist for over one hour and given the failure of mechanical ventilation to provide adequate oxygenation, the decision was to start VV-ECMO.

The patient’s left femoral vein was cannulated with a 28-Fr heparin-coated cannula for blood return and the left internal jugular vein was cannulated with a 21-Fr cannula for blood access. The patient was placed on VV-ECMO (CentriMag [Thoratec, Pleasanton, CA, USA] short-term external circulatory device) with initial settings of flow 4.5 liters per minute, speed 3,300 revolutions per minute, and ECMO FiO_2_ 100%, sweep three liters per minute. She was started on heparin drip, received infusion of neuromuscular blocking agent cisatracurium for a short period, and then remained sedated with hydromorphone and lorazepam. She continued to have episodes of fevers up to 101°F, and repeated sputum cultures demonstrated the presence of Acinetobacter species and Proteus mirabilis, while blood cultures were negative. She was placed on cefepime for ventilator-associated pneumonia and the episodes of fevers gradually improved.

On the following two weeks, the patient was gradually able to wean down VV-ECMO settings, decreasing ECMO FiO_2_ to 40%, and flow to 3.6 liters per minute. On hospital day 20, she had a transvenous pacemaker placed due to several episodes of bradycardia with pauses lasting up to 19 seconds during oral care, likely vagally mediated. On hospital day 21, a bronchoscopy was performed and showed minimal secretions and no mucus plug. Given her prolonged time intubated, she underwent tracheostomy placement on hospital day 22. On hospital day 26, the patient was finally able to have VV-ECMO explanted, after 18 days of extracorporeal therapy. As she continued to improve clinically, the mechanical ventilation was successfully weaned off on hospital day 38. The patient was noted to have no neurological deficits, and was able to be downgraded from ICU to the medical ward on the following day. 

## Discussion

COVID-19 infection was declared a pandemic by the World Health Organization on March 11, 2020, and has raised numerous concerns given its high hospital admission rate of approximately 15% of patients, with 5% being considered critically ill [1]. Patients admitted to the ICU with severe COVID-19 pneumonia often require mechanical ventilation for acute hypoxic respiratory failure, but the role of ECMO in the management of these cases is still unclear to date.

VV-ECMO has been used for ARDS to correct hypoxia and hypercarbia in patients with refractory disease despite optimized mechanical ventilation (using a lung-protective strategy), prone ventilation, and neuromuscular blockade [2]. The extracorporeal therapy permits ultraprotective strategies on mechanical ventilation, therefore decreasing the risk of ventilator-induced lung injury while allowing the pulmonary parenchyma to recover [3].

Postmortem analysis of patients with COVID-19 induced lung injury has shown bilateral diffuse alveolar damage and desquamation of pneumocytes associated with pulmonary edema, lesions consistent with ARDS, hence supporting the theory that ECMO could be beneficial for this group of patients [4]. Moreover, ECMO has also been proposed as rescue therapy for patients with Middle East respiratory syndrome caused by another coronavirus (MERS-CoV) who failed conventional strategies [5].

The current World Health Organization interim guideline considers referring critically ill patients with COVID-19 infection to centers able to provide ECMO therapy [6]. Unfortunately, the early publications reporting the use of ECMO for patients with COVID-19 infection have demonstrated a high mortality rate. Observational studies from China showed a mortality rate of approximately 82.3% (14 of 17 patients undergoing ECMO therapy died) [7]. In a retrospective case series of 1,591 consecutive patients admitted to the ICU with COVID-19 infection in Italy, only five patients were placed on ECMO, but information about the mortality in these patients was not provided as some of them were still in the ICU at the time of publication, limiting the interpretation of the study [8].

Additionally, the complexity of ECMO therapy requires a well-trained multidisciplinary team present only at expert centers, and the high resource cost represents a challenge to the health care system in times of a global pandemic. As of May 28, 2020, a cumulative total of more than 5,491,000 COVID-19 cases have been reported around the world, with more than 349,190 deaths [9]. The COVID-19 pandemic rapidly overwhelmed health care systems, making the decision to offer ECMO especially harder as it could mean diverting resources from other patients.

Limitations related to VV-ECMO therapy also include increased risk of thrombosis, limited role as cardiac support, and high risk of infection in caregivers due to its inevitably high demand of maintenance. When VV-ECMO is being considered, we believe that dual-site cannulation should be preferred for COVID-19 patients, as the cannulation can be safely performed at bedside without the need for fluoroscopy or transesophageal echocardiography, consequently requiring fewer personal to come into the room.

Considering the limitations of this resource-intensive therapy, clinical judgment is crucial to decide whether ECMO is an appropriate option for the patient. To date, there is no published study or guideline for selection criteria; therefore, each ECMO center is required to develop individual center-specific inclusion and exclusion criteria. The risk-to-benefit ratio needs be determined on a case-by-case basis, and priority should be given to younger patients, preferably with no significant comorbidities and higher survival chances, with likely short-term need for ECMO.

In the present case, our patient met these criteria, as she was 32 years old with no significant comorbidities except for obesity. The Respiratory ECMO Survival Prediction score was 4 points, with an expected in-hospital survival chance of 74%. While on ECMO, we aimed for lung-protective mechanical ventilation settings with pressure control mode, low PEEP, peak pressure around 30-35 cm H_2_O, and FiO_2_ 40%. The supportive medical management also included high doses of intravenous steroids, a five-day course of azithromycin and hydroxychloroquine, one dose of tocilizumab, and transfusion with convalescent plasma which are mostly used at this point in a compassionate basis for treatment of severe COVID-19 and cytokine release syndrome associated with it. We also aggressively treated lung congestion with spot doses of intravenous furosemide, and fortunately her renal function improved quickly, with good urine output throughout the hospital course. As we already expected, she had a prolonged course intubated and eventually required tracheostomy placement. However, considering her very poor oxygenation level before the VV-ECMO placement, we strongly believe that this was a life-saving therapy. 

## Conclusions

It is important to recognize the significant limitations of VV-ECMO therapy for patients being treated for SARS-CoV-2 and, given the lack of guidelines on this subject, the centers are required to determine their own inclusion and exclusion criteria. Nevertheless, for young patients with no significant underlying conditions who are critically ill despite optimized mechanical ventilation and medical management, we believe that extracorporeal therapy could represent a reasonable option when available. 
